# MRI radiomics captures early treatment response in patient-derived organoid endometrial cancer mouse models

**DOI:** 10.3389/fonc.2024.1334541

**Published:** 2024-05-07

**Authors:** Heidi Espedal, Kristine E. Fasmer, Hege F. Berg, Jenny M. Lyngstad, Tomke Schilling, Camilla Krakstad, Ingfrid S. Haldorsen

**Affiliations:** ^1^Department of Clinical Medicine, University of Bergen, Bergen, Norway; ^2^Mohn Medical Imaging and Visualization Centre, Department of Radiology, Haukeland University Hospital, Bergen, Norway; ^3^Western Australia National Imaging Facility, Centre for Microscopy, Characterization and Analysis, University of Western Australia, Perth, WA, Australia; ^4^Centre for Cancer Biomarkers, Department of Clinical Science, University of Bergen, Bergen, Norway; ^5^Department of Gynecology and Obstetrics, Haukeland University Hospital, Bergen, Norway

**Keywords:** patient-derived organoids, MRI radiomics, endometrial cancer, preclinical imaging, patient-derived model

## Abstract

**Background:**

Radiomics can capture microscale information in medical images beyond what is visible to the naked human eye. Using a clinically relevant mouse model for endometrial cancer, the objective of this study was to develop and validate a radiomic signature (*RS*) predicting response to standard chemotherapy.

**Methods:**

Mice orthotopically implanted with a patient-derived grade 3 endometrioid endometrial cancer organoid model (O-PDX) were allocated to chemotherapy (combined paclitaxel/carboplatin, n=11) or saline/control (n=13). During tumor progression, the mice underwent weekly T2-weighted (T2w) magnetic resonance imaging (MRI). Segmentation of primary tumor volume (vMRI) allowed extraction of radiomic features from whole-volume tumor masks. A radiomic model for predicting treatment response was derived employing least absolute shrinkage and selection operator (LASSO) statistics at endpoint images in the orthotopic O-PDX (*RS_O*), and subsequently applied on the earlier study timepoints (*RS_O* at baseline, and week 1-3). For external validation, the radiomic model was tested in a separate T2w-MRI dataset on segmented whole-volume subcutaneous tumors (*RS_S*) from the same O-PDX model, imaged at three timepoints (baseline, day 3 and day 10/endpoint) after start of chemotherapy (n=8 tumors) or saline/control (n=8 tumors).

**Results:**

The *RS_O* yielded rapidly increasing area under the receiver operating characteristic (ROC) curves (AUCs) for predicting treatment response from baseline until endpoint; AUC=0.38 (baseline); 0.80 (week 1), 0.85 (week 2), 0.96 (week 3) and 1.0 (endpoint). In comparison, vMRI yielded AUCs of 0.37 (baseline); 0.69 (w1); 0.83 (week 2); 0.92 (week 3) and 0.97 (endpoint). When tested in the external validation dataset, *RS_S* yielded high accuracy for predicting treatment response at day10/endpoint (AUC=0.85) and tended to yield higher AUC than vMRI (AUC=0.78, p=0.18). Neither *RS_S* nor vMRI predicted response at day 3 in the external validation set (AUC=0.56 for both).

**Conclusions:**

We have developed and validated a radiomic signature that was able to capture chemotherapeutic treatment response both in an O-PDX and in a subcutaneous endometrial cancer mouse model. This study supports the promising role of preclinical imaging including radiomic tumor profiling to assess early treatment response in endometrial cancer models.

## 1 Introduction

Endometrial cancer is the most common gynecological cancer in high-income countries and the incidence is increasing, primarily due to growing societal obesity and ageing populations (1, 2). The primary treatment for endometrial cancer is surgery which is curative in most patients with low-risk, early-stage disease. Albeit, about 15% of endometrial cancer patients experience recurrence and have a poor prognosis (3–5).

Adjuvant chemotherapy, using a combination of paclitaxel and carboplatin, is recommended in patients with high-risk histology (endometrioid grade 3 and non-endometrioid endometrial carcinoma) or advanced stage disease (6). Unfortunately, chemotherapy only moderately improves the survival in endometrial cancer patients and causes adverse side-effects (7–9). Furthermore, evaluation of treatment response is routinely carried out by imaging, weeks to months after start of the treatment, and is presently based on changes in tumor size or appearance of new metastases. Earlier prediction of treatment response may allow prompt treatment modifications and reduce unnecessary side-effects affecting the quality of life in non-responders (7).

Pelvic magnetic resonance imaging (MRI) is a key part of the clinical management of endometrial cancer, and is recommended for preoperative local staging (10). In the past few years, MRI radiomic tumor profiling has been introduced, promoting non-invasive markers of tumor heterogeneity from whole-volume primary tumors. Radiomic tumor profiling is based on computational extraction of large numbers of quantitative imaging features that inherently characterize tumor tissue and tumor microenvironment. Commonly derived radiomic features describe tumor shape (i.e. size, circularity, spicularity), and tumor texture (i.e. higher order statistics quantifying spatial distribution of pixel/voxel intensity values in the region of interest) (11). MRI radiomic models have been reported to predict response to chemotherapy in breast cancer and osteosarcoma (12, 13), chemoradiotherapy in gliomas and rectal cancer (14, 15), and immunotherapy in brain metastases (16). In endometrial cancer patients, MRI radiomics has been shown to predict aggressive disease and is thus promising for serving as a supplement in preoperative risk assessment (17–19). In preclinical models of pancreatic cancer, MRI radiomic profiling has been shown to predict treatment response (20, 21). To our knowledge, no former study has explored whether radiomics from preclinical endometrial cancer models may be used to predict treatment response.

Preclinical models represent a vital tool for developing and accessing new therapeutic strategies in endometrial cancer (22). Recently, an organoid-based patient-derived xenograft mouse models (O-PDX) that recapitulate the histopathology- and genetic profiles of the donor tumor tissue has been developed (23). Furthermore, clinically relevant imaging methods including preclinical MRI to quantify tumor progression and assess treatment response in these models as been established (24). The development of non-invasive tools for early detection of treatment response is essential for rapid adaptation of treatment strategies in non-responding endometrial cancer patients. In this study, we explore the temporal change in MRI-derived tumor volume (vMRI) and MRI tumor radiomic features in orthotopically implanted O-PDX mice, allocated to either chemotherapy or no treatment (control group). We aim to develop a radiomic signature (*RS*) predicting treatment response to chemotherapy and to assess whether the *RS* enables early prediction of treatment response in the orthotopic O-PDX model. Finally, we aim to validate the *RS* in an independent subcutaneous O-PDX model from the same O-PDX model.

## 2 Materials and methods

### 2.1 Animal model

All animal experiments were conducted in accordance with Norwegian and European regulations (approval ID 20194, Mattilsynet) an Association for Assessment and Accreditation of Laboratory Animal Care (AAALAC)-approved facility. Hysterectomy tumor specimen donated by one consenting patient (approval ID 2015/2333 and 2018/548, REK vest) diagnosed with grade 3, endometrioid endometrial cancer, and International Federation of Gynecology and Obstetrics (FIGO) stage IIIC1 was used to establish a patient-derived organoid model (23). Organoids were immersed 1:1 in matrigel and orthotopically implanted (2x10^6^ cells) into the left uterine horn in n=24 female NOD/SCID IL2rγ^null^ (NSG) mice to generate the mouse model (O-PDX) as previously described (25). The mice were monitored for disease symptoms including lethargy, ataxia and weight loss following a scoring system, and were sacrificed when the score met the pre-determined threshold or at the end of the study.

### 2.2 Study design

**Figure 1:**
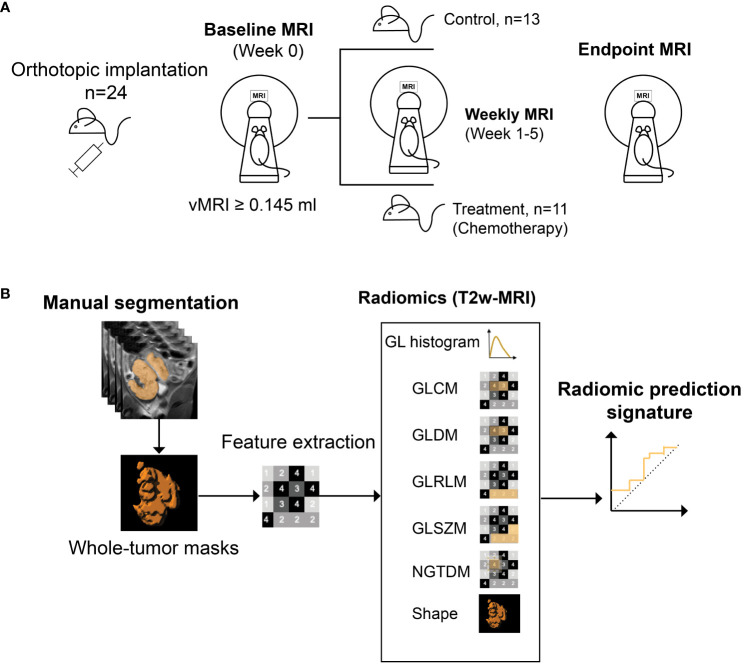
Study design. Mice (n=24) with orthotopically implanted patient-derived organoids, underwent weekly MRI from 21 days post implantation **(A)**. When tumor volume (vMRI) segmented on T2 weighted (T2w)-MRI reached a threshold of ≥0.145 ml (defined as baseline/week 0), mice were either allocated to chemotherapy- (combined carboplatin/paclitaxel) (n=11) or control (saline) (n=13) groups. MRI scanning was continued weekly during week 1-5, until euthanization/endpoint (varied from week 3-5 for the individual mice). Radiomic features were extracted from all timepoints from baseline to endpoint, using manually segmented whole-volume tumor masks from the T2w images **(B)**. A radiomic signature (*RS*) predicting treatment groups (chemotherapy vs. control) was generated at endpoint using least absolute shrinkage and selection operator (LASSO) statistics. GL, grey level; GLCM, GL co-occurrence matrix; GLDM, GL dependence matrix; GLRLM, GL run length matrix; GLSZM, GL size zone matrix, NGTDM, neighboring gray tone difference matrix.

The orthotopically implanted mice (n=24) underwent weekly MRI from 21 days post-implantation until euthanization by cervical dislocation. When MRI-measured tumor volume (vMRI) reached ~0.145 ml, the mice were allocated into treatment- (n=11) or control groups (n=13) (**Figure 1A**). A small to moderate tumor size of approximately 0.150 ml at baseline balances growth rate and treatment sensitivity in our model. The allocation timepoint was defined as the baseline/week 0 (a detailed timeline is given in **Supplementary Table 1**). The treatment group received intraperitoneal injections of combined carboplatin (15 mg/kg) and paclitaxel (12 mg/kg), according to the current international guideline for management of endometrial cancer (6). The control group received saline (100 μl) intraperitoneally. The first treatment/saline injections were given immediately after the baseline MRI (week 0), and then continued twice a week for both groups throughout the study.

### 2.3 MR imaging and tumor segmentation

T2-weighted (T2w) images were acquired on a preclinical 7 Tesla MRI scanner (PharmaScan, Bruker Biospin, Ettlingen, Germany) using a mouse body quadrature volume resonator in a single-coil configuration. Mice were anesthetized by sevoflurane (2.5%) mixed in oxygen and respiration and body temperature were monitored during scanning. The T2w images were acquired coronally with TE/TR 25/2500 ms, 5 averages, 160x160 matrix, 32x32 mm field of view, 1 mm slice thickness and 0.2x0.2 mm resolution. Choice of MRI sequence was based on previous finding (24), showcasing T2w-imaging as a robust MRI method for tumor visualization and tumor delineation in orthotopic endometrial cancer models. We did not include any T1-weighted sequences due to intravenous contrast agent toxicity in mice, especially considering the significant number of imaging time points in the study. Segmentation of vMRI were conducted for all timepoints, by manually delineating the tumor borders slice by slice, covering the entire tumor, using the open-source software ITK-SNAP (Version 3.8) (26). To assess interreader agreement, vMRI segmentations were performed independently by two readers, with >10 years and ~3 months experience in preclinical MRI reading, respectively. The masks from the most experienced reader were used for further radiomic analyses.

### 2.4 Radiomic feature extraction and model selection

T2w images with corresponding tumor volume masks were imported into the software application Radiomics Frontier (Siemens Healthineers, Erlangen, Germany), which is based on the open source, image biomarker standardization initiative (IBSI (27))-compliant PyRadiomics library (28). Prior to feature extraction, all images were resampled to an isotropic voxel size of 1 mm using a Gaussian interpolator and bin width set to 20. In total, 110 radiomic features were extracted from each tumor at all timepoints; 18 grey level (GL) histogram features; 24 GL co-occurrence matrix (GLCM) features; 14 GL dependence matrix (GLDM) features; 16 GL run-length matrix (GLRLM) features; 16 GL size zone matrix (GLSZM) features; 5 neighboring grey tone difference matrix (NGTDM) features; and 17 shape features (**Figure 1B**). Using the extracted radiomic features at study endpoint (varying from week 3-5 for individual mice), a radiomic signature (*RS*) predicting treatment groups (chemotherapy vs. control) was constructed using logistic least absolute shrinkage and selection operator (LASSO) statistics (29). The regularization parameter (λ) was optimized by 5-fold cross validation, and the *RS* was constructed by a linear combination of the LASSO selected features (f1-f6, **Table 1**) multiplied by their respective model coefficients (RS, **Table 1**). The performance of the *RS* for predicting treatment groups/response (all mice in the treatment group were classified as responders) was further evaluated at all study timepoints (baseline, week 1-3, endpoint) in the orthotopic model (*RS_O*).

**Table 1:** Radiomic features (f1-f6) selected by least absolute shrinkage and selection operator (LASSO) for prediction of treatment response (chemotherapy vs. control) at endpoint in the orthotopic mouse model. A radiomic signature (*RS*) for predicting treatment response was constructed by linear combination of LASSO model coefficients and f1-f6.

Radiomic features	LASSO model coefficients
f1 (TotalEnergy)	0.406
f2 (GLCM_IDMN)	0.412
f3 (GLCM_MCC)	0.174
f4 (GLRLM_RunEntropy)	1.030
f5 (Shape_LeastAxisLength)	0.205
f6 (Shape_VoxelVolume)	0.085
Model constant	0.240
RS = (0.406*f1)+(0.412*f2)+(0.174*f3)+(1.030*f4)+(0.205*f5)+(0.085*f6)+0.240	

f, radiomic feature; GLCM, grey level co-occurrence matrix; GLRLM, grey level run length matrix; IDMN, inverse difference moment normalized; MCC, Matthew’s correlation coefficient.

### 2.5 Histological analyses

Animals were euthanized immediately following the last imaging timepoint (endpoint). Uterine tumors were formalin fixed and embedded in paraffine before tissue slice sections (5 μm) were cut. Sections were stained with standard hematoxylin and eosin (HE) or subjected to immunohistochemistry for detection of Ki-67 expression. Briefly, for Ki-67 staining sections were dewaxed with xylene and rehydrated in ethanol before microwave antigen retrieval in target retrieval solution, pH 6. Following peroxidase block, the sections were incubated for 1 hour at room temperature with rabbit monoclonal antibody to Ki-67 (1:100; Abcam, ab16667) followed by 30 min of incubation with secondary HRP-conjugated anti-rabbit antibody and 5-8 min with DAB-chromogen (EnVision detection system, Dako). Sections were counterstained in hematoxylin before dehydration and mounting.

Stained tissue slides were scanned at 20X magnification using a slide scanner (VS120, Olympus). Automatic quantification of tumor cells (HE) and proportion (%) of Ki-67 positive cells was performed using the open-source QuPath software (V0.2.0) using 1-3 regions of interest (number depending on tumor size) placed in representative areas of the tumor.

### 2.6 Validation dataset

The *RS* for predicting treatment groups in the orthotopic mouse model was further validated in an independent imaging dataset of subcutaneous tumors from the same O-PDX model. The mice in the validation cohort underwent longitudinal imaging at a 7 Tesla MRI system (DRYMAG 7017, MR Solutions, Guildford, United Kingdom) and started an identical treatment protocol (combined carboplatin/paclitaxel or control/saline) as in the orthotopic model when tumor size (vMRI) reached >0.07 ml (baseline). Four mice with bilateral tumors were randomized to treatment (n=8 tumors) and four mice to control (n=8 tumors). Further details on the subcutaneous O-PDX model are given in **Supplementary Method Description** and **Supplementary Figure 2**.

T2w-MRI was acquired at baseline (prior to treatment), early treatment (day 3) and endpoint (day 10). The T2w images were further used for whole-volume tumor segmentations and radiomic feature extraction utilizing the same software application and software settings as for the orthotopic model. The *RS* developed in the orthotopic model (**Table 1**) was applied for prediction of treatment groups (chemotherapy vs. control) and evaluated at the different imaging time points (*RS_S* at baseline, early and endpoint) in this separate and independent subcutaneous O-PDX study.

### 2.7 Statistical analyses

Intraclass correlation coefficients (ICCs) from a two-way random effects model were used to assess interreader agreement for measuring vMRI, with the tumors being segmented by two different readers in both the orthotopic and subcutaneous model. The readers were blinded to study groups. Differences in vMRI, tumor cell density and Ki-67 expression between the groups were assessed using a Student’s t- or Mann–Whitney test following normality assessment by the Shapiro–Wilk test. The *RS* for prediction of treatment groups was constructed using LASSO statistics. The penalty parameter (λ) was selected by 5-fold cross-validation and minimization of the cross-validation function. *RS_O*, *RS_S* and vMRI were compared for prediction of treatment group using area under the receiver operating characteristics curves (AUCs) and DeLongs’ test of equality. Weekly change in the radiomic signature features was assessed by calculating fold change normalized to baseline values (Δchange) (Microsoft Excel), and weekly differences were tested by Mann-Whitney tests. Correlations between vMRI, tumor cell density, Ki-67 and *RS_O* and the radiomic features incorporated in the *RS*, were assessed using Spearman’s rank order correlation test. Statistical analyses were conducted in STATA 17.0 (StataCorp, College Station, TX) and GraphPad Prism v9 (GraphPad Software, San Diego, California USA). Reported p-values were generated by two-sided tests and considered significant when <0.05.

## 3 Results

### 3.1 Orthotopic tumor progression in the treatment/control groups

**Figure 2:**
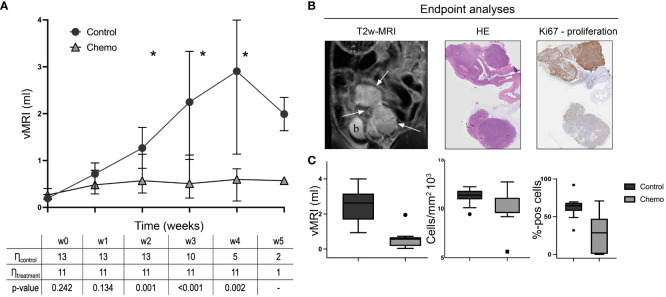
Orthotopic tumor progression in the two groups (chemotherapy vs control). MRI-derived tumor volumes (vMRI, median ± 95% confidence interval) in mice allocated to chemotherapy (carboplatin/paclitaxel, n=11) or control groups (saline, n=13) **(A)**. All mice with the orthotopically implanted patient-derived organoids (O-PDX) were imaged weekly from baseline (w0) until euthanization/endpoint (w3-w5 for individual mice). From w2-w4, the median vMRI was significantly lower in the chemotherapy group compared to the controls (*p ≤ 0.002, Mann-Whitney test, w5 not tested because of group size) **(A)**. The O-PDX models exhibit characteristic imaging findings with hyperintense tumors (arrows; b, bladder) on T2w-MRI coronal series (a representative tumor is shown at endpoint) **(B)**. At endpoint, tumor tissue from all mice were stained using hematoxylin and eosin (HE) for tumor cell density quantification, and immunohistochemistry for Ki-67 expression **(C)**. Median vMRI (ml), tumor cell density (cells/mm^2^) and tumor Ki67 (%-positive cells) at endpoint were significantly lower in the chemotherapy- compared to the control group (p ≤ 0.03, Mann-Whitney test).

All the orthotopically implanted O-PDX mice developed intrauterine tumors visible at MRI after 21 days. The tumors were hyperintense with distinct tumor boundaries on T2w-MRI (**Figure 2B**). At baseline (start of treatment), vMRI was similar in the two study groups (chemotherapy vs. control) (p=0.2, **Supplementary Figure 1A**). The control group had increasing vMRI from baseline to endpoint (median vMRI=0.19 ml (baseline/w0); 0.71 ml (week 1/w1); 1.27 ml (w2); 2.25 ml (w3); 2.90 ml (w4) and 1.99 ml (w5)), whereas the treatment group had relatively stable vMRI throughout the study (median vMRI=0.27 ml (baseline); 0.48 ml (w1); 0.57 ml (w2); 0.51 ml (w3); 0.60 ml (w4) and 0.57 ml (w5)) (**Figure 2A**). From week 2, the median vMRI was significantly lower in the mice receiving chemotherapy vs. control (p ≤ 0.002, w2-w4). In week 4, three mice with large tumors were euthanized, explaining the apparent decline of median vMRI from week 4 (n=5 mice) to week 5 (n=2) in the control group (**Figure 2A**; **Supplementary Figure 1B**). Individually, all tumors in the control group had increasing volumes from baseline to endpoint/euthanasia (**Supplementary Figure 1B**).

At endpoint, ranging from week 3-5 after baseline for the individual mice (**Supplementary Table 1**), median vMRI was significantly lower in the chemotherapy group compared to the control group (p<0.001, **Figure 2C**). Cell density (from HE-staining) and proliferation scores (from Ki67 immunohistochemistry) confirmed treatment effect at the cellular level with significantly lower median cell density (p=0.03) and median proliferation (p<0.001) in the chemotherapy versus the control group at endpoint (**Figure 2C**).

### 3.2 Radiomic signature predicts treatment response in the orthotopic model

The interreader agreement for vMRI segmentation in the orthotopic model was excellent with an ICC of 0.98 (95% confidence interval (CI):0.97-0.99) (**Supplementary Table 2**). From the endpoint data set, six radiomic features (f1-f6) were selected by LASSO and used to construct a *RS* predicting treatment (chemotherapy vs. control); four of these were texture features (f1-f4) and two were shape features (f5-f6) (**Table 1**).

**Figure 3:**
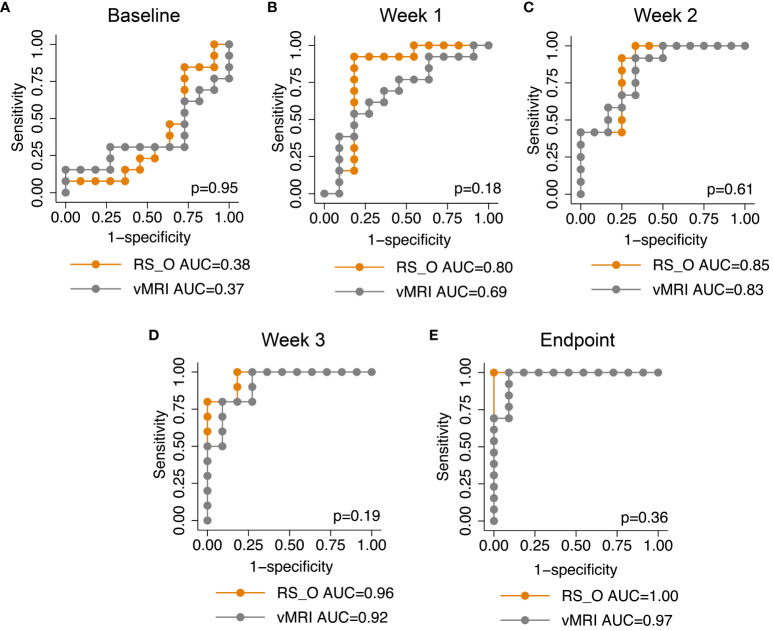
The radiomic signature and longitudinal area under the receiver operating characteristic curves. The radiomic signature (*RS*) generated at endpoint for predicting treatment (chemotherapy vs. control) in the orthotopic mouse model (*RS_O*), yielded high area under the receiver operating characteristic curves (AUCs) from one week after start of treatment (week1: AUC=0.80; **B**). During the treatment course, *RS_O* yielded increasing AUCs of 0.85 at week 2 **(C)**, 0.96 at week 3 **(D)** and 1.00 at endpoint **(E)**. In comparison, MRI derived tumor volume (vMRI) yielded lower AUCs of 0.69 at week 1 **(B)**, 0.83 at week 2 **(C)**, 0.92 at week 3 **(D)** and 0.97 at endpoint **(E)** (p>0.05 for all, DeLongs’ test of equality).

Applied at endpoint (model construction timepoint), the *RS* yielded an excellent prediction of treatment groups, i.e., treatment response (*RS_O* AUC=1.00), although not significantly higher than tumor volume (vMRI AUC=0.97, p=0.36) (**Figure 3E**). At baseline, no difference was expected between the treatment and control groups, which was confirmed by low AUCs for both *RS_O* (AUC=0.38) and vMRI (AUC=0.37) (**Figure 3A**). However, already at w1, *RS _O* yielded high accuracy for prediction of treatment (AUC=0.80), which tended to be higher than that of vMRI (AUC=0.69, p=0.18) (**Figure 3B**). In w2 and w3 the AUCs were further increasing for both *RS_O* (AUC=0.85 (w2) and 0.96 (w3)) and vMRI (AUC=0.83 (w2) and 0.92 (w3)) (**Figures 3C, D**).

### 3.3 Delta radiomics

**Figure 4:**
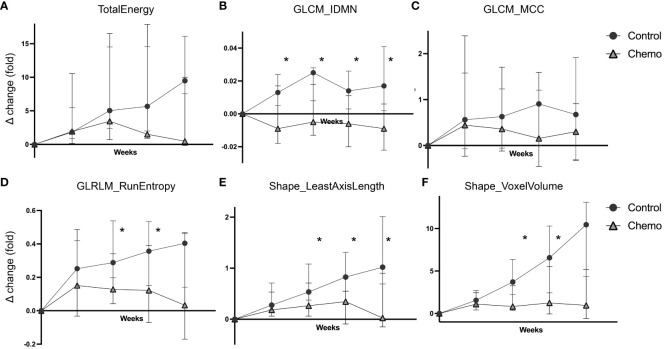
Delta radiomics in the chemotherapy vs the control group. Temporal changes in the radiomic values (weekly fold change relative to baseline, Δchange, median ± 95% confidence interval) plotted separately for the two treatment groups (chemotherapy vs. control) in the orthotopic O-PDX mouse model. Six radiomic features were included in the RS signature; TotalEnergy **(A)**, GLCM_IDMN **(B)**, GLCM_MCC **(C)**, GLRLM_RunEntropy **(D)**, Shape_LeastAxisLenght **(E)** and Shape_VoxelVolume **(F)**. For GLCM_IDMN, GLRLM_RunEntropy, Shape_LeastAxisLength and ShapeVoxelVolume **(B, D-F)** the control group exhibited significantly increased Δchange compared to the chemotherapy group (*p ≤ 0.03, Mann-Whitney test) from week 2. GLCM_IDMN, grey level co-occurrence matrix inverse difference moment normalized; GLCM_MCC, grey level co-occurrence matrix Matthew’s correlation coefficient; GLRLM, grey level run length matrix.

The temporal change in the radiomic values (Δchange) from baseline to endpoint for the features included in the *RS* (**Table 1**) is depicted according to treatment groups (chemotherapy vs. control) in **Figure 4**. The texture feature *GLCM_IDMN*, showed significantly higher Δchange in the control group compared to the chemotherapy group already at w1 (p=0.007), and throughout the study (p ≤ 0.01, w2-w4) (**Figure 4B**). *GLRLM_RunEntropy* (**Figure 4D**), and the two shape features *Shape_LeastAxisLength* (**Figure 4E**) and *Shape_VoxelVolume* (**Figure 4F**), showed significantly higher Δchange in the control group in w2 (p ≤ 0.03 for all), in w3 (p ≤ 0.01 for all), and for *Shape_LeastAxisLength*, also in w4 (p=0.006, **Figure 4E**).

### 3.4 Correlations between radiomics, tumor volume and proliferation marker Ki-67

Correlations between the model-selected features (f1-f6) and corresponding tumor volumes (vMRI) at each timepoint (baseline/w0-w4) are presented in **Table 2**. There was an increasing moderate to strong correlation (ρ) between vMRI and the radiomic features *TotalEnergy* (ρ=0.59 (w0) – 0.94 (w4), p ≤ 0.003), *GLRLM_RunEntropy* (ρ=0.57 (w0) – 0.94 (w4), p ≤ 0.004) and *Shape_LeastAxisLength* (ρ=0.69 (w0) – 0.96 (w4), p ≤ 0.002) throughout the study. *GLCM_IDMN* was only weakly-to-moderately correlated to vMRI from week 1 and onwards (ρ=0.43 (w1) – 0.62 (w4), p ≤ 0.04), and *GLCM_MCC* only moderately correlated to vMRI at w3 (ρ=0.49, p=0.02).

**Table 2:** Correlation coefficients (ρ)^a^ between MRI-assessed tumor volume (vMRI) and the individual features (f1-f6) included in the radiomic signature at baseline/w0-w4 in the orthotopic model (*RS_O*) for chemotherapy- and control groups combined.

	f1, ρ *(TotalEnergy)*	f2, ρ *(GLCM_IDMN)*	f3, ρ *(GLCM_MCC)*	f4, ρ *(GLRLM_RunEntropy)*	f5, ρ *(Shape_LeastAxisLength)*	f6, ρ *(Shape_VoxelVolume)*
vMRI w0	0.59*	0.26	0.31	0.57*	0.69*	0.85**
vMRI w1	0.70*	0.43*	0.38	0.66*	0.74**	0.90**
vMRI w2	0.88**	0.64*	0.39	0.85**	0.77**	0.97**
vMRI w3	0.92**	0.67*	0.49*	0.84**	0.92**	0.99**
vMRI w4	0.94**	0.62*	0.38	0.94**	0.96**	1.0**

a Spearman rank correlation, 0.05≤p≤0.0001* and p<0.0001** marked by italic Week 0-2, n=24; Week 3, n=22; Week 4, n=16.

f, radiomic feature; GLCM, grey level co-occurrence matrix; GLRLM, grey level run length matrix; IDMN, inverse difference moment normalized; MCC, Matthew’s correlation coefficient.

At endpoint, there was a strong positive correlation between *RS_O* and vMRI (ρ=0.90, p<0.001). Further, *RS_O* showed moderate positive correlations with both cell density (ρ=0.44 [p=0.03]) and Ki-67 expression (ρ=0.55 [p=0.005]), while vMRI only showed a moderate positive correlation with Ki-67 expression (ρ=0.51 [p=0.01]) (**Table 3**). Individually, the radiomic features *TotalEnergy*, *GLRLM_RunEntropy*, *Shape_LeastAxisLength* and *Shape_VoxelVolume* were all highly positively correlated to vMRI (ρ≥0.85; p<0.001), while *GLCM_IDMN* and *GLCM_MCC* were moderately correlated to vMRI (ρ=0.72 and 0.53, respectively; p ≤ 0.006) (**Table 3**).

**Table 3:** Correlation (ρ)^a^ at endpoint in the orthotopic model, between vMRI, tumor cell density (cells/mm^2^), Ki67 (%-pos cells), the radiomic signature (*RS_O*) and the six features included in the *RS_O* signature (f1-f6).

	vMRI (ρ)	Cell density (ρ)	Ki67 (ρ)
Cell density (cells/mm^2^)	0.35	*-*	*-*
Ki-67 (%-pos cells)	0.51*	0.32	*-*
RS_O	0.90**	0.44*	0.55*
f1 (TotalEnergy)	0.89**	0.43*	0.52*
f2 (GLCM_IDMN)	0.72**	0.43*	0.50*
f3 (GLCM_MCC)	0.53*	0.19	0.35
f4 (GLRLM_RunEntropy)	0.85**	0.42*	0.64*
f5 (Shape_LeastAxisLength)	0.85**	0.34	0.48*
f6 (Shape_VoxelVolume)	0.95**	0.35	0.49*

a Spearman’s rank correlation, 0.05≤p≤0.0001* and p<0.0001** marked by italic.

f, radiomic feature; GLCM, grey level co-occurrence matrix; GLRLM, grey level run length matrix; LASSO, least absolute shrinkage and selection operator; IDMN, inverse difference moment normalized; MCC, Matthew’s correlation coefficient.

### 3.5 Radiomic signature predicts treatment response in the validation model

**Figure 5:**
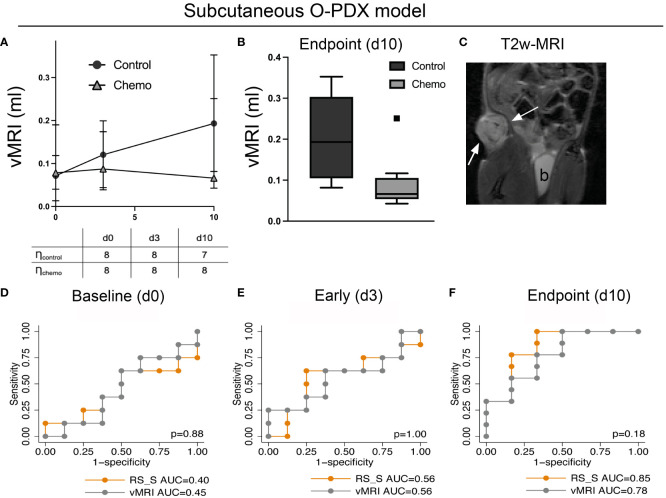
Validation dataset. The radiomic signature (*RS*) for predicting treatment response (chemotherapy vs. control) developed at endpoint in the orthotopic model was validated in a separate imaging dataset (*RS_S*) utilizing the same patient-derived organoid model grown subcutaneously. The mice in this independent study underwent an identical treatment regime as the mice with orthotopically implanted tumors. T2w-MRI was performed at baseline (day 0), at an early timepoint (day 3) and at endpoint (day 10) **(A, C)**. T2w-MRI depicting a representative tumor (arrows; b, bladder) at day 3 **(C)**. At endpoint, MRI-assessed median tumor volume (vMRI) was significantly lower in the chemotherapy group than in the control group (p=0.01) **(B)**, and vMRI yielded an area under the receiver operating characteristic curve (AUC) of 0.78 for predicting treatment groups whereas *RS_S* yielded an AUC of 0.85 **(F)**. At the earlier timepoints, neither vMRI nor *RS_S* was able to predict treatment groups in the subcutaneous model **(D, E)**. p>0.05 for all, DeLongs’ test of equality.

To validate the *RS* developed in the orthotopic model, we utilized T2w images from an independent imaging dataset (acquired on a different 7 T MRI system) of the same O-PDX model (from the same patient) grown subcutaneously (**Supplementary Method Description**; **Figure 5**). Similar to the orthotopic model, the interreader agreement for vMRI measurements was excellent for the subcutaneous tumors (ICC [95% CI]: 0.93 [0.88-0.96]) (**Supplementary Table 2**).

At endpoint, the median vMRI was significantly lower in the chemotherapy (median vMRI=0.07 ml) versus the control group (median vMRI=0.19 ml, p=0.01, **Figures 5A, B**). There was no significant difference in vMRI between the groups at baseline (median vMRI=0.07 and 0.08 ml for the control- and chemotherapy group, respectively) or at early timepoint (day 3; median vMRI=0.12 and 0.09 ml for the control- and chemotherapy group, respectively. p≥0.38 for both). The *RS*, when applied in the subcutaneous model (*RS_S*) yielded high AUC for prediction of treatment response (chemotherapy vs. control group) at endpoint (AUC=0.85), which tended to be higher than the AUC for vMRI (AUC=0.78, p=0.18) (**Figure 5F**). At the early timepoint (day 3), neither *RS_S* nor vMRI was able to predict response (AUC=0.56 for both) (**Figure 5E**). As for the orthotopic model, the treatment and control groups were expected to be similar at baseline in the subcutaneous model - which was confirmed by low baseline AUC for both *RS_S* and vMRI (AUC=0.44 and 0.45, respectively) (**Figure 5D**).

## 4 Discussion

This study describes temporal changes in MRI radiomic tumor features in a preclinical treatment-sensitive O-PDX endometrial cancer mouse model stratified to chemotherapy or no therapy. We present an MRI radiomic signature yielding increasingly high accuracy for predicting treatment response after start of therapy. Interestingly, the radiomic signature tended to predict treatment response earlier than vMRI, already one week after start of treatment, suggesting that distinct changes in radiomic features, may parallel and even precede treatment effect on tumor volume. The MRI radiomic signature also yielded high accuracy for predicting treatment response in mice with subcutaneous tumors from the same O-PDX endometrial cancer model. This indicates that treatment-induced changes in radiomic tumor features are similar across O-PDX implantation sites (orthotopic vs. subcutaneous). Importantly, the radiomic tumor features associated with treatment response in this preclinical study, may represent promising markers for clinical translation and further testing, to provide early prediction of chemotherapeutic response in patients with advanced endometrial cancer.

In patients with metastatic- or recurrent endometrial cancer in whom non-surgical treatment (i.e., chemo- radio – or hormonal therapy) is the mainstay of treatment, there is an immediate need to develop robust and non-invasive imaging tools for early prediction of therapeutic response. The preclinical O-PDX model used in this study (23) showed excellent response to combined carboplatin/paclitaxel treatment, with overall decreasing or stabilized tumor volumes following treatment, while untreated tumors had an explosive tumor growth. The derived MRI radiomic tumor signatures predicted treatment groups with increasingly high accuracy from week 1 to endpoint. At endpoint, tumor tissue analyses confirmed treatment effect at the cellular level with significantly lower median cell density (p=0.03) and median proliferation/Ki-67 scores (p<0.001) in the chemotherapy- versus the control group. Using a similar MRI radiomics methodology, Eresen et al. (20) showed that MRI radiomic features predicted early therapeutic response following dendritic cell vaccination in a preclinical pancreatic ductal adenocarcinoma model. The derived tumor radiomic features were investigated in relation to different histological tumor markers, including Ki-67 expression. In line with our study, Ki-67 was shown to be lower in the treatment group compared with the control group at endpoint. Eresen and colleagues also reported a strong positive correlation (Pearson r=0.84) between Ki-67 expression and one of the derived radiomic features (*mean vertical wavelet coefficient)*. In the present study, moderate positive correlations between Ki-67 and the radiomic signature features (f1-f6) were observed, with strongest correlation (Spearman ρ=0.65) between Ki-67 and *GLRLM_RunEntropy* (f4). Importantly, both studies demonstrate intriguing associations between radiomic tumor features at the mesoscopic scale, and immunohistochemical tumor markers at the microscopic scale. Radiomic tumor profiling may thus bridge the gap between imaging markers and microscopic tumor markers and complement subjective conventional diagnostic reading (e.g. reporting of tumor size) in the assessment of treatment response and tailoring of therapy.

In recent years, several studies have linked tumor radiomics from primary tumor depicted by preoperative MRI and computed tomography to clinical phenotype and patient outcome in endometrial cancer (17, 30, 31). Radiomics and radiogenomics applications may thus support personalized treatment in the management of endometrial cancer (32, 33). However, less is known about radiomics as a potential tool for predicting treatment response in endometrial cancer patients receiving non-surgical treatment. In the present study we have utilized a clinically relevant O-PDX mouse model, undergoing weekly MRI scanning during treatment (chemotherapy vs. control), and derived a radiomic tumor signature that predicts treatment response with high accuracy already at one week after start of treatment (AUC=0.80, week 1, **Figure 3B**). The radiomic signature also predicts response in an independent validation cohort (subcutaneous model) (AUC=0.85, day 10, **Figure 5F**). Importantly, several of the radiomic features incorporated in the signature are either derivatives of tumor shape/volume (f5-f6; ρ(vMRI)≥0.85 for both) or strongly positively correlated to tumor volume (f1, f4; ρ≥0.85 for both). Nevertheless, the remaining two features *GLCM_IDMN* (f2) and *GLCM_MCC* (f3), were only weakly -to- moderately correlated to tumor volume (f2: ρ=0.72; f3: ρ=0.53) Interestingly, *GLCM_IDMN* was significantly higher in the controls compared to the chemotherapy group already one week after treatment start, and *GLCM_IDMN* and tumor volume were not significantly correlated at baseline. At endpoint, *GLCM_IDMN* also showed a moderate positive correlation both to cell density (ρ=0.43) and to cell proliferation/Ki-67 (ρ=0.50). Overall, this supports that *GLCM_IDMN* is a relatively volume-independent radiomic marker, reflecting mesoscale tumor features that are closely linked to established microscopic features, and that that *GLCM_IDMN* can capture early chemotherapeutic response.

### 4.1 Limitations

Although radiomics has shown promising results in numerous scientific studies for a large variety of purposes, radiomic feature values are prone to variabilities due to, amongst others, differences in scanner models, image processing and image analyses (34). Also, there is an overall lack of validation of promising radiomic features in separate and independent study cohorts. In preclinical studies, it is considered good ethical practice to restrict the number of animals used (35). With a limited number of animals (typically no more than 10) and a large number of radiomic features (hundreds to thousands), it is challenging to develop prediction models without any overfitting bias. In the present study we have applied state-of-the-art LASSO statistics, for radiomic feature reduction and prediction modelling. The radiomic signature yielded excellent performance for prediction of treatment response in the orthotopic model at endpoint (AUC=1.00), although with an inherent risk of model overfitting. However, when applied in an independent subcutaneous model cohort, scanned on a separate MRI system, the high prediction performance of the radiomic signature was reproduced (AUC=0.85), making it unlikely that overfitting has substantially biased the results. The radiomic signature was developed, tested and validated in animals treated with chemotherapy. The performance of the signature for predicting response to other types of therapies including immunotherapy and targeted therapies remains unanswered.

## 5 Conclusions

This study demonstrates the potential of MRI radiomics to predict early treatment response in a clinically relevant O-PDX endometrial cancer mouse model The same radiomic signature predicted treatment response in a subcutaneous PDX endometrial cancer mouse model, supporting its transferability across tumor sites. Further validation in independent preclinical studies and subsequent testing of radiomic profiling in the clinic are needed, to define its potential value for early prediction of therapeutic response and tailoring of endometrial cancer treatment.

## Data availability statement

The raw data supporting the conclusions of this article will be made available by the authors, without undue reservation.

## Ethics statement

The studies involving humans were approved by Regional komité for forskningsetikk, Vest Norge (Rek vest). The studies were conducted in accordance with the local legislation and institutional requirements. The participants provided their written informed consent to participate in this study. The animal study was approved by Mattilsynet (The Norwegian Food Authority). The study was conducted in accordance with the local legislation and institutional requirements.

## Author contributions

HE: Conceptualization, Data curation, Formal analysis, Investigation, Writing – original draft, Writing – review & editing. KF: Conceptualization, Formal analysis, Investigation, Writing – original draft, Writing – review & editing. HB: Data curation, Resources, Writing – review & editing. JL: Data curation, Writing – review & editing. TS: Formal analysis, Writing – review & editing. CK: Funding acquisition, Project administration, Resources, Writing – review & editing. IH: Conceptualization, Funding acquisition, Project administration, Supervision, Writing – original draft.

## References

[1] Sung H Ferlay J Siegel RL Laversanne M Soerjomataram I Jemal A. Global cancer statistics 2020: GLOBOCAN estimates of incidence and mortality worldwide for 36 cancers in 185 countries. CA Cancer J Clin. (2021) 71(3):209–49. doi: 10.3322/caac.21660 33538338

[2] Gu B Shang X Yan M Li X Wang W Wang Q. Variations in incidence and mortality rates of endometrial cancer at the global, regional, and national levels, 1990–2019. Gynecol Oncol. (2021) 161:573–80. doi: 10.1016/j.ygyno.2021.01.036 33551200

[3] Lu KH Broaddus RR. Endometrial cancer. N Engl J Med. (2020) 383:2053–64. doi: 10.1056/NEJMra1514010 33207095

[4] Amant F Moerman P Neven P Timmerman D Van Limbergen E Vergote I. Endometrial cancer. Lancet. (2005) 366:491–505. doi: 10.1016/S0140-6736(05)67063-8 16084259

[5] Fung-Kee-Fung M Dodge J Elit L Lukka H Chambers A Oliver T. Follow-up after primary therapy for endometrial cancer: a systematic review. Gynecol Oncol. (2006) 101:520–9. doi: 10.1016/j.ygyno.2006.02.011 16556457

[6] Concin N Matias-Guiu X Vergote I Cibula D Mirza MR Marnitz S. ESGO/ESTRO/ESP guidelines for the management of patients with endometrial carcinoma. Int J Gynecol Cancer. (2021) 31:12–39. doi: 10.1136/ijgc-2020-002230 33397713

[7] Matei D Filiaci V Randall ME Mutch D Steinhoff MM DiSilvestro PA. Adjuvant chemotherapy plus radiation for locally advanced endometrial cancer. N Engl J Med. (2019) 380:2317–26. doi: 10.1056/NEJMoa1813181 PMC6948006 31189035

[8] Galaal K Al Moundhri M Bryant A Lopes AD Lawrie TA. Adjuvant chemotherapy for advanced endometrial cancer. Cochrane Database Syst Rev. (2014) 2014:Cd010681. doi: 10.1002/14651858.CD010681.pub2. 24832785 PMC6457820

[9] de Boer SM Powell ME Mileshkin L Katsaros D Bessette P Haie-Meder C. Adjuvant chemoradiotherapy versus radiotherapy alone for women with high-risk endometrial cancer (PORTEC-3): final results of an international, open-label, multicentre, randomised, phase 3 trial. Lancet Oncol. (2018) 19:295–309. doi: 10.1016/S1470-2045(19)30395-X 29449189 PMC5840256

[10] Oaknin A Bosse TJ Creutzberg CL Giornelli G Harter P Joly F. Endometrial cancer: ESMO Clinical Practice Guideline for diagnosis, treatment and follow-up☆. Ann Oncol. (2022) 33:860–77. doi: 10.1016/j.annonc.2022.05.009 35690222

[11] Lambin P Leijenaar RTH Deist TM Peerlings J de Jong EEC van Timmeren J. Radiomics: the bridge between medical imaging and personalized medicine. Nat Rev Clin Oncol. (2017) 14:749–62. doi: 10.1038/nrclinonc.2017.141 28975929

[12] McAnena P Moloney BM Browne R O’Halloran N Walsh L Walsh S. A radiomic model to classify response to neoadjuvant chemotherapy in breast cancer. BMC Med Imaging. (2022) 22:225. doi: 10.1186/s12880-022-00956-6 36564734 PMC9789647

[13] Bouhamama A Leporq B Khaled W Nemeth A Brahmi M Dufau J. Prediction of histologic neoadjuvant chemotherapy response in osteosarcoma using pretherapeutic MRI radiomics. Radiology: Imaging Cancer. (2022) 4:e210107. doi: 10.1148/rycan.210107 36178349 PMC9530773

[14] Zhang Z He K Wang Z Zhang Y Wu D Zeng L. Multiparametric MRI radiomics for the early prediction of response to chemoradiotherapy in patients with postoperative residual gliomas: an initial study. Front Oncol. (2021) 11. doi: 10.3389/fonc.2021.779202 PMC8636428 34869030

[15] Song M Li S Wang H Hu K Wang F Teng H. MRI radiomics independent of clinical baseline characteristics and neoadjuvant treatment modalities predicts response to neoadjuvant therapy in rectal cancer. Br J Cancer. (2022) 127:249–57. doi: 10.1038/s41416-022-01786-7 PMC9296479 35368044

[16] Bhatia A Birger M Veeraraghavan H Um H Tixier F McKenney AS. MRI radiomic features are associated with survival in melanoma brain metastases treated with immune checkpoint inhibitors. Neuro-Oncology. (2019) 21:1578–86. doi: 10.1093/neuonc/noz141 PMC7145582 31621883

[17] Fasmer KE Hodneland E Dybvik JA Wagner-Larsen K Trovik J Salvesen Ø. Whole-volume tumor MRI radiomics for prognostic modeling in endometrial cancer. J Magnetic Resonance Imaging. (2021) 53:928–37. doi: 10.1002/jmri.27444 PMC7894560 33200420

[18] Chen J Gu H Fan W Wang Y Chen S Chen X. MRI-based radiomic model for preoperative risk stratification in stage I endometrial cancer. J Cancer. (2021) 12:726–34. doi: 10.7150/jca.50872 PMC7778535 33403030

[19] Yan BC Li Y Ma FH Feng F Sun MH Lin GW. Preoperative assessment for high-risk endometrial cancer by developing an MRI- and clinical-based radiomics nomogram: A multicenter study. J Magnetic Resonance Imaging. (2020) 52:1872–82. doi: 10.1002/jmri.27289 32681608

[20] Eresen A Yang J Shangguan J Li Y Hu S Sun C. MRI radiomics for early prediction of response to vaccine therapy in a transgenic mouse model of pancreatic ductal adenocarcinoma. J Transl Med. (2020) 18:61. doi: 10.1186/s12967-020-02246-7 32039734 PMC7011246

[21] Eresen A Yang J Shangguan J Benson AB Yaghmai V Zhang Z. Detection of immunotherapeutic response in a transgenic mouse model of pancreatic ductal adenocarcinoma using multiparametric MRI radiomics: A preliminary investigation. Acad Radiol. (2021) 28:e147–54. doi: 10.1016/j.acra.2020.04.026 PMC7704817 32499156

[22] Moiola CP Lopez-Gil C Cabrera S Garcia A Van Nyen T Annibali D. Patient-derived xenograft models for endometrial cancer research. Int J Mol Sci. (2018) 19(8):2431. doi: 10.3390/ijms19082431 30126113 PMC6121639

[23] Berg HF Hjelmeland ME Lien H Espedal H Fonnes T Srivastava A. Patient-derived organoids reflect the genetic profile of endometrial tumors and predict patient prognosis. Commun Med. (2021) 1:1–14. doi: 10.1038/s43856-021-00019-x 35602206 PMC9053236

[24] Espedal H Berg HF Fonnes T Fasmer KE Krakstad C Haldorsen IS. Feasibility and utility of MRI and dynamic 18F-FDG-PET in an orthotopic organoid-based patient-derived mouse model of endometrial cancer. J Trans Med. (2021) 19:406. doi: 10.1186/s12967-021-03086-9 PMC8474962 34565386

[25] Haldorsen IS Popa M Fonnes T Brekke N Kopperud R Visser NC. Multimodal imaging of orthotopic mouse model of endometrial carcinoma. PloS One. (2015) 10:e0135220. doi: 10.1371/journal.pone.0135220 26252891 PMC4529312

[26] Yushkevich PA Piven J Hazlett HC Smith RG Ho S Gee JC. User-guided 3D active contour segmentation of anatomical structures: significantly improved efficiency and reliability. Neuroimage. (2006) 31:1116–28. doi: 10.1016/j.neuroimage.2006.01.015 16545965

[27] Zwanenburg A Vallières M Abdalah MA Aerts HJWL Andrearczyk V Apte A. The image biomarker standardization initiative: standardized quantitative radiomics for high-throughput image-based phenotyping. Radiology. (2020) 295:328–38. doi: 10.1148/radiol.2020191145 PMC7193906 32154773

[28] van Griethuysen JJM Fedorov A Parmar C Hosny A Aucoin N Narayan V. Computational radiomics system to decode the radiographic phenotype. Cancer Res. (2017) 77:e104–7. doi: 10.1158/0008-5472.CAN-17-0339 PMC5672828 29092951

[29] Tibshirani R. Regression shrinkage and selection via the lasso. J R Stat Soc Ser B (Methodological). (1996) 58:267–88. doi: 10.1111/j.2517-6161.1996.tb02080.x

[30] Ytre-Hauge S Salvesen ØO Krakstad C Trovik J Haldorsen IS. Tumour texture features from preoperative CT predict high-risk disease in endometrial cancer. Clin Radiol. (2021) 76:79.e13–20. doi: 10.1016/j.crad.2020.07.037 32938538

[31] Ytre-Hauge S Dybvik JA Lundervold A Salvesen ØO Krakstad C Fasmer KE. Preoperative tumor texture analysis on MRI predicts high-risk disease and reduced survival in endometrial cancer. J Magnetic Resonance Imaging. (2018) 48:1637–47. doi: 10.1002/jmri.26184 30102441

[32] Hoivik EA Hodneland E Dybvik JA Wagner-Larsen KS Fasmer KE Berg HF. A radiogenomics application for prognostic profiling of endometrial cancer. Commun Biol. (2021) 4:1363. doi: 10.1038/s42003-021-02894-5 34873276 PMC8648740

[33] Di Donato V Kontopantelis E Cuccu I Sgamba L Golia D’Augè T Pernazza A. Magnetic resonance imaging-radiomics in endometrial cancer: a systematic review and meta-analysis. Int J Gynecol Cancer. (2023) 33:1070–6. doi: 10.1136/ijgc-2023-004313 37094971

[34] Moskowitz CS Welch ML Jacobs MA Kurland BF Simpson AL. Radiomic analysis: study design, statistical analysis, and other bias mitigation strategies. Radiology. (2022) 304:265–73. doi: 10.1148/radiol.211597 PMC9340236 35579522

[35] Fenwick N Griffin G Gauthier C. The welfare of animals used in science: How the “Three Rs” ethic guides improvements. Can Vet J. (2009) 50:523–30. PMC2671878 19436640

