# Supplementary material for: MRI radiomics captures early treatment response in patient-derived organoid endometrial cancer mouse models
Source: Front Oncol. 2024 May 7;14:1334541. doi: 10.3389/fonc.2024.1334541 (PMC11106402; doi:10.3389/fonc.2024.1334541)
Supplement: Supplementary file 1 [file DataSheet_1.docx]

Supplementary material

| **Supplementary Table 1**: Overview of timeline and study scheme in the orthotopic mouse model, from organoid implantation (day 1-2) until euthanization by cervical dislocation (varying from day 39-55 for the individual mice). When MRI-assessed tumor volume (vMRI) reached a threshold of 0.145 ml, the mice were allocated to chemotherapy- or control groups (inclusion time/baseline). All mice were scanned weekly with MRI. | | | | | |
| --- | --- | --- | --- | --- | --- |
| **Time after implantation**  (Days) | **Action** | **Inclusion**  Control-group (n) | **Inclusion** Treatment-group (n) | **Baseline**^a^,  total (n) | **Euthanization**  (post-MRI) |
| 1-2^b^ | Orthotopic implant: 24 mice | - | - |  | - |
| ~21^c^ | MRI, n=24 | 7 | 1 | 8 | - |
| ~28 | MRI, n=24 | 1 | 10 | 11 | - |
| ~35 | MRI, n=24 | 4 | - | 4 | 2 (controls) |
| ~42 | MRI, n=22 | 1 | - | 1 | - |
| ~49 | MRI, n=22 | - | - | - | 6 (controls) |
| ~56^d^ | MRI, n=16 | - | - | - | 16 (5 controls, 11 treatment)^e^ |
| ^a^The manuscript refers to baseline as week 0, and following weeks as week 1-5. ^b^Orthotopic implantation was carried out over 2 days. ^c^MRI was performed on Mondays or Tuesday. Treatment injections (carboplatin/paclitaxel or saline) were given immediateley following MRI (Monday/Tuesday) and the following Thursday/Friday. ^d^The study was ended when local covid-19 protocols recommended terminating all animal experiments at the earliest time after reaching statistically significant results, not permitting survival studies. ^e^All remaining animals were euthanized at this timepoint: 5/13 (38%) control animals and 11/11 (100%) treatment animals | | | | | |

# Supplementary Table 1

**
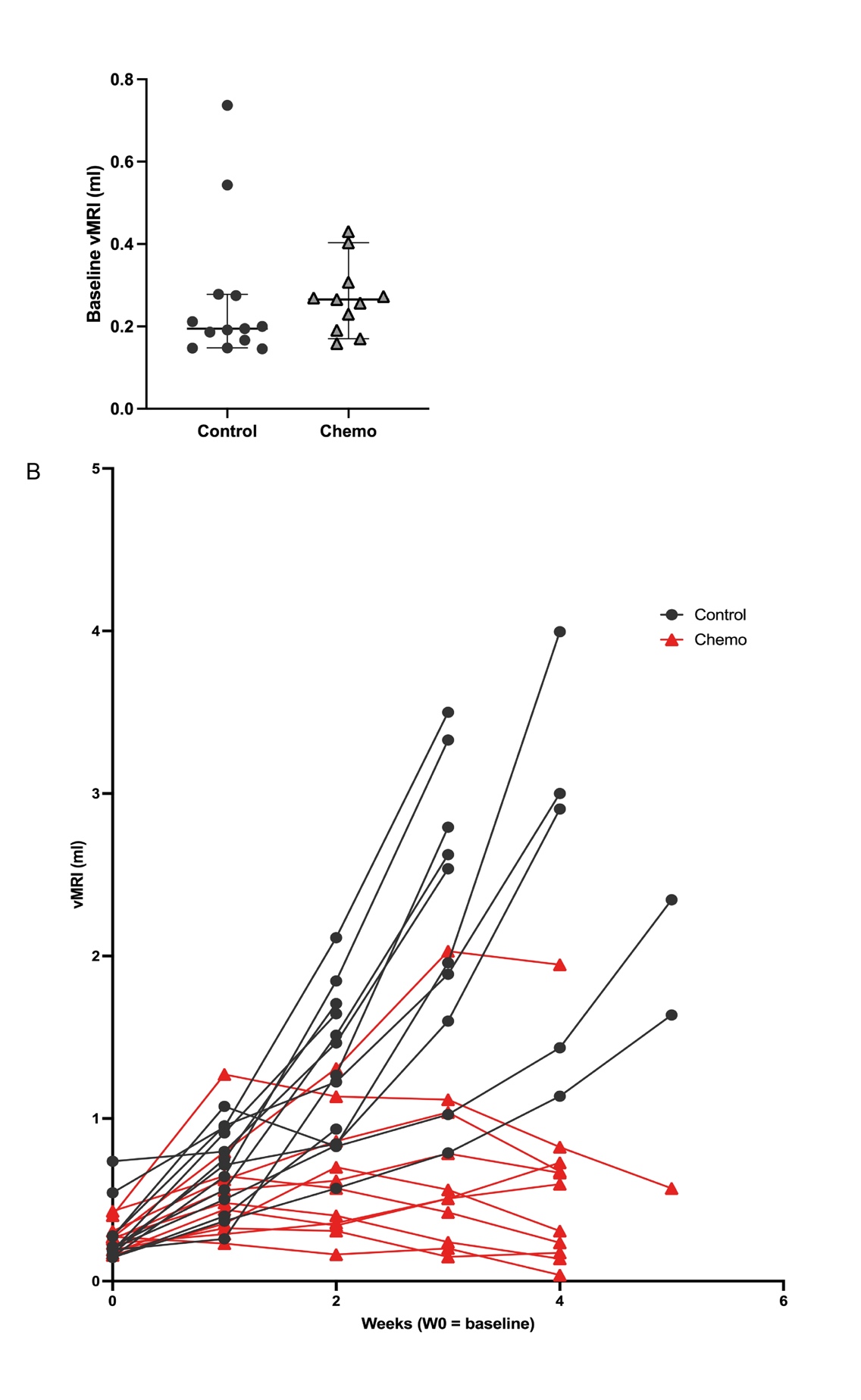
**

**Supplementary Figure 1:** Longitudinal tumor growth in the orthotopic model depicted for all mice in the chemotherapy (red)- and control (black) groups. At baseline (week 0), MRI assessed tumor volumes (vMRI) were similar for mice in the chemotherapy- and control groups (p=0.2) (A). From baseline to endpoint, all mice in the control group had an explosive increase in vMRI, while mice in the chemotherapy group had overall stable or only a slight increase in vMRI (B).

**Supplementary Table 2:**

Intraclass correlation coefficients (ICC, two-way random-effects model) for tumor volume measured by MRI (vMRI) assessed by two independent readers.

| Tumor model | ICC (95% confidence interval) | *n*, (tumors/series) |
| --- | --- | --- |
| Orthotopic | 0.98 (0.97-0.99) | 112 |
| Subcutaneous | 0.93 (0.88-0.96) | 47 |

**Supplementary method description: Validation dataset**

NOD/SCID IL2rγ^null^ (NSG) mice (n=8) were subcutaneously injected with patient-derived organoids, O-PDX (0.5*10^6^ cells immersed 1:1 in matrigel) derived from the same patient as for the orthotopic model. The cells were injected bilaterally in the dorsal flank under sevoflurane anesthesia (2.5 % in oxygen) and tumor growth was monitored biweekly by palpation and caliper measurement. Mice were randomly allocated into treatment- (n=4, 8 tumors total) and control (n=4, 8 tumors total) groups when the largest tumor was >0.07 ml (vMRI). The treatment regimen was the same (drugs, administration, dose and frequency) as for the orthotopic model. The first intraperitoneal injections (chemotherapy or saline) were given immediately after the baseline MRI scan. At the early timepoint (day 3) the mice had received one injection/treatment cycle and at the endpoint three cycles had been given (S Fig. 2).

Images were acquired on a preclinical 7 Tesla MRI scanner (DRYMAG 7017, MR Solutions, Guildford, United Kingdom) in a dual-mouse bed using a 65 mm diameter volume coil optimized for imaging multiple mice. Mice were anesthetized by sevoflurane (2.5 %) mixed in oxygen and respiration and body temperature were monitored during scanning. T2-weighted images were acquired coronally (TE/TR 45/3500 ms, 3 averages, matrix 239x256, field of view 30x60 mm, slice thickness 0.9 mm, resolution 0.25x0.24 mm). Manual whole-volume tumor segmentation, post-processing and feature extraction were performed identical to that for the orthotopic model. It should be noted that for statistical testing all tumors we considered as individual tumors, although comprising pairs of tumors in eight different mice.

**Supplementary Figure 2**

**
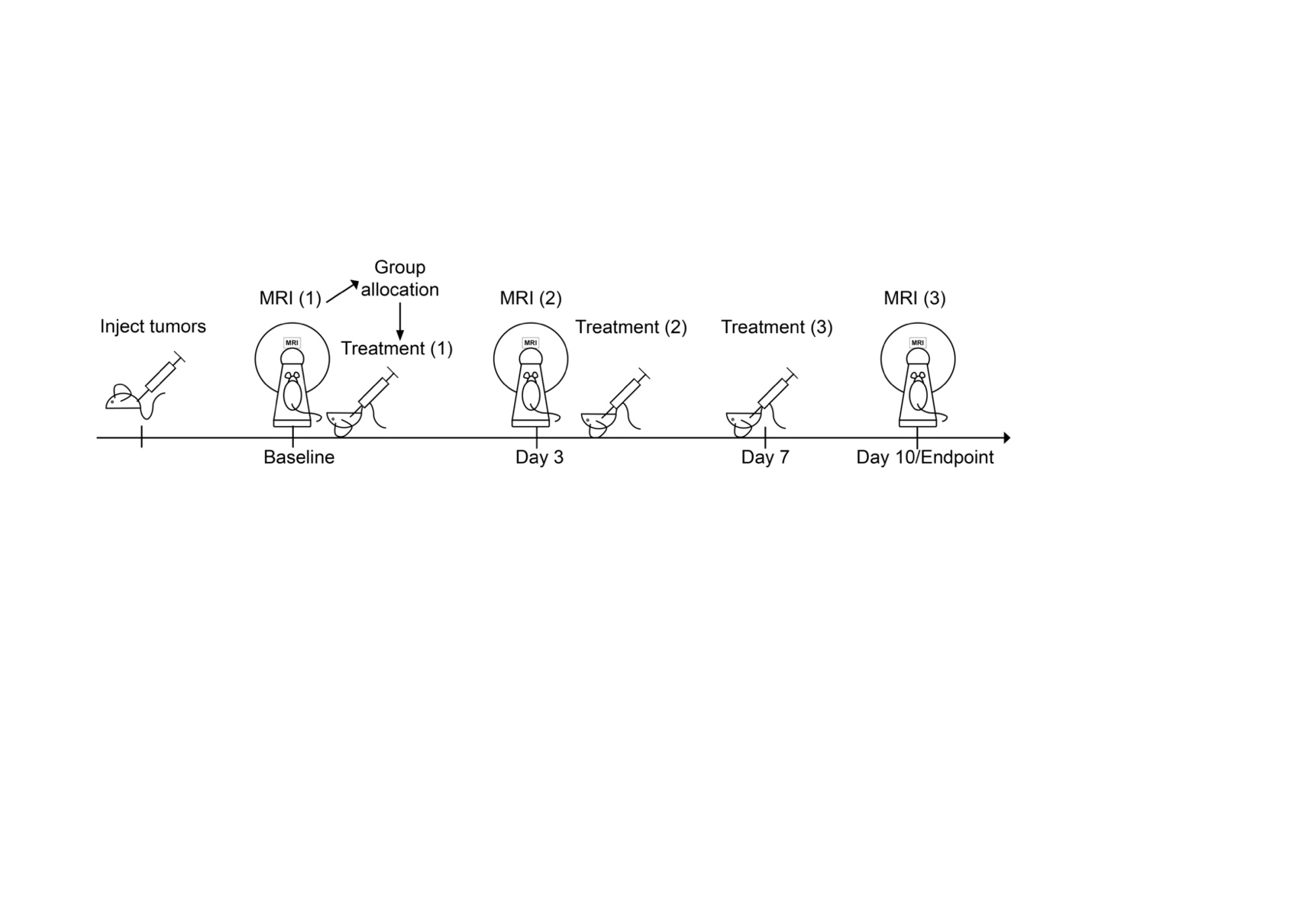
**

Timeline of the subcutaneous study. The mice were imaged at three timepoints; baseline, day 3 (early) and day 10 (endpoint). The first treatment (saline/control or combined paclitaxel/carboplatin) was given immediately following the baseline MRI.
